# Traditional, complementary and alternative medical systems and their contribution to personalisation, prediction and prevention in medicine—person-centred medicine

**DOI:** 10.1186/1878-5085-3-15

**Published:** 2012-11-06

**Authors:** Paolo Roberti di Sarsina, Mauro Alivia, Paola Guadagni

**Affiliations:** 1 High Council of Health, Ministry of Health, Rome, 00144, Italy; 2 Charity Association for Person Centred Medicine, Via Siepelunga, 36/12, Bologna, 40141, Italy; 3 Observatory and Methods for Health, University of Milano-Bicocca, Milan, 20126, Italy; 4 Italian Society of Anthroposophic Medicine (SIMA), Milan, 20121, Italy

**Keywords:** Traditional, Complementary and alternative medicine, Biomedicine, Person-centred medicine, Personalised medicine, Prediction, Prevention, Salutogenesis, Health-care reform, Health-care education, Therapeutic relationship

## Abstract

*Traditional, complementary and alternative medical* (TCAM) s*ystems* contribute to the foundation of *person-centred medicine* (PCM), an epistemological orientation for medical science which places the *person* as a physical, psychological and spiritual entity at the centre of health care and of the therapeutic process. PCM wishes to broaden the bio-molecular reductionistic approach of medical science towards an integration that allows people, doctors, nurses, health-care professionals and patients to become the real protagonists of the health-care scene. The doctor or caregiver needs to act out of empathy to meet the unique value of each human being, which unfolds over the course of a lifetime from conception to natural death. Knowledge of the human being should not be instrumental to economic or political interests, ideology, theories or religious dogma. Research needs to be broadened with methodological tools to investigate person-centred medical interventions. Salutogenesis is a fundamental principle of PCM, promoting health and preventing illness by strengthening the individual's self-healing abilities. TCAM systems also give tools to predict the insurgence of illness and treat it before the appearance of overt organic disease. A task of PCM is to educate people to take better care of their physical, psychological and spiritual health. Health-care education needs to be broadened to give doctors and health-care workers of the future the tools to act in innovative and highly differentiated ways, always guided by deep respect for individual autonomy, personal culture, religion and beliefs.

## Review

### The need for person-centred medicine

Patients themselves demand an improvement in the quality of medical interventions with greater humanisation, personalisation of treatments and adequate information received in a safe environment to be able to make choices about their therapeutic process freely [1]. They want a doctor who will talk to them, listen to what they say and give them advice about how to get better and protect their health in the future. They want to be given the time and the space to express during the consultation, and once a therapeutic relationship is established, they wish to continue seeing the same person to give continuity to the process of healing. In many cases, the wish for a prescription is secondary to the wish of being cared for [2].

Many doctors and caregivers already practise *person-centred medicine* (PCM) with growing interest from colleagues and institutions. There is a perceived need to create a more satisfying therapeutic relationship, individualising treatments beyond clinical guidelines to suit the whole person in the context of his or her bio-psycho-spiritual biography [3]. PCM takes on the task to rebuild an effective therapeutic relationship based on trust, empathy, compassion and responsiveness to individual needs and values. To become individualised, diagnosis and treatment need to take into consideration the human being in his or her full expression [4]. The central question in PCM is: how can we restore the integrity, the dignity and the sacred and ethical value of the human being as a bio-psycho-spiritual entity? This raises the further question: how can we develop a concept of the ‘whole’ if only the physico-chemical forces acting in the organism are considered real and amenable to investigation by scientific research? How can we investigate the psychological and spiritual realities of the human being? How can we go beyond a dualistic view? PCM is a concept that is becoming increasingly used in medical education, in primary care and in other fields of health care where there is a need to reintegrate the analytical and fragmented image of the human being provided by specialism [4,5].

Biomedicine, the dominant western medical approach, is based on a mechanistic model of the human being, which stems from Virchow's theory of the cell as the unit of life. Life is considered to be no more than cellular activity ruled by physico-chemical laws. ‘Living organisms appeared to me like moved bodies, their only difference from inorganic bodies being their tendency to form cells, but always with mechanical movements.’ ‘If up until now it has been impossible to produce the origin of life starting from purely physical and chemical laws, it seems to me that every reasonable physiologist who admits to an origin before life, cannot look for it in anything other than the combined action of physico-chemical forces’ [6].

Virchow's thinking erased the traditional philosophy from which Hippocratic and Galenic medicine had developed in Europe and were continued by the medieval monastic tradition and further developed by Paracelsus. This was based on a holistic, sacred view of the human being based on principles which we now call salutogenesis, resilience, sense of coherence, internal and external sustainability, personal responsibility, self-regard and individual value, in other words, person-centred medicine. The Greek concept of the four humours was the result of a profound reflection more than of rational investigation [7].

However, the time came to move away from this, towards a ‘scientific vision [that] no longer consists of religious faith and philosophical transcendence’. ‘The knowledge of laws is quite sufficient; investigating the foundations of the law is a form of transcendent conceit’ [8]. This has allowed the development of analytical thinking but in the main, only what is demonstrable and reproducible, linked by a mechanical cause-and-effect model, is considered truthful and scientific. There is no science outside this. Current biomedicine, which has developed from this model, is responsible for undeniable advances. Cells, organelles, physiological and chemical reactions, pathophysiological and biochemical processes, DNA and genetic codes, and more recently systems biology are studied in great detail, giving us a huge body of knowledge and therapeutic interventions developed as a consequence of these discoveries, but this is no longer enough. Life is more than the result of measurable biochemical reactions [9]. Thoughts, feelings and cognitively based actions are more than the result of physico-chemical processes. How can we not lose the human being? How can we broaden our concepts of science and medicine to investigate and heal the human being as a whole in a scientific way?

### Traditional, complementary and alternative medicine and personalisation, prevention and prediction in medicine

We can find answers to these questions in *traditional, complementary and alternative medical* (TCAM) *systems* which are used by 80% of people in the so-called developing world, by 360 million people in China and by around 150 million citizens and 300,000 registered health-care professionals in Europe [7,10].

What is traditional, alternative and complementary medicine?

• TCAM is a term used to represent a variety of different medical systems and health care methods that stem from European culture and from other philosophical backgrounds and traditions.

• They are based on the knowledge, skills and practices used to protect and restore health.

• They aim to prevent, diagnose and treat physical or mental illness and include medication and non-medication therapies.

• They share a vision of the human being as a unique physical, psychological and spiritual entity where, as Aristotle put it, ‘the whole is more than the sum of its parts’.

• Within this holistic view, it is the physiological or pathological interaction between these aspects that determines health or illness. The genesis of health or illness also depends on the interaction between the human being, nature and the cosmos.

• TCAM systems are based on salutogenetic principles. Patients perceive this generation of health as a growing feeling of wellbeing, consequent to the stimulation of innate self-healing abilities through the adoption of a healthier lifestyle and the medication and non-medication therapies specific to each TCAM system [11].

*Traditional Chinese medicine* (TCM) is founded on the principles that preserving health is the best approach to disease prevention, it is better to reinforce our body's health before the insurgence of illness rather than having to cure it once it has developed and it is better to regulate lifestyles and nutrition regimes before the development of disease rather than having to prescribe treatments once problems have arisen. The integration between mind and body is essential. The body (*Xing*) is seen as the material substrate of mental activity, and the mind (*Shen*) is the governor of the body. One cannot do without the other.

In *traditional Tibetan medicine* (TTM), the human being is seen as body, mind and energy. The mind and the three great mental poisons of anger, attachment and mental obtuseness are essential both for wellbeing and health. The mind and the five elements are represented by three humours that are the quintessence of the energy that constantly flows into the human body and maintains health and mental alertness. Both TCM and TTM have a complex therapeutic system that includes plant-based remedies, forms of physical therapies such as massage and acupuncture, baths, forms of meditation and ritual movement (Yoga or Tai Chi). They are examples of a multifaceted therapeutic system that can address the human being as a bio-psycho-spiritual whole, with an intrinsically salutogenetic approach to disease prevention and healing [12].

In *Ayurvedic medicine*, life is seen as the continuous interaction between the body, sense organs, mind, soul and a living being in continuous interaction and adaptation between sensory perception, mental elaboration and adaptive response towards the environment. The core aim of Ayurveda is to prevent illness, look after health, maintain health and promote longevity. To this aim, topical and systemic therapeutic options are individualised according to the three Ayurvedic principles (Dosha): *Kapha*, *Pitta* and *Vata*, the articulated expressions of matter which govern the bio-psycho-spiritual functions of the human being. The individual human being is characterised by the unique combination of the three Dosha that make up his or her individual constitution which influences not only the bio-psycho-spiritual characteristics of a person, but also the predisposition towards certain diseases and states of imbalance. A constitutional assessment can guide primary prevention and also give the diagnostic and therapeutic tools to identify a pathological tendency in its initial stages, before it becomes established with symptoms or overt organic pathology [13].

Other TCAM systems also allow these forms of personalisation, prediction and prevention, each from their point of view. All TCAM systems are holistic; they relate physical symptoms to all other aspects of the human being, his or her natural and social environment; they share the common element of being person-centred. These systems are based on an understanding of health that is intrinsically and ontologically connected to the person in its entirety, as an individual, inseparable in body, psyche and spirit, which includes all behavioural, psychological, spiritual, environmental and cultural aspects. With the danger of being simplistic, we could say that biomedicine has developed a militaristic vision based on focusing on disease in various parts of the body, localising it and eliminating it using technologies and treatments that can be necessary and life-saving but unfortunately economically inaccessible to a large proportion of the world's population. TCAM systems have developed a therapeutic continuum with concepts of prevention, philosophically and ecologically developed on maintaining health, on the local ecosystem as a source of medicines, on food as medicine, on the importance of the caregiver-patient relationship as a therapeutic tool, on ‘taking care of the person’ in the long term in a more sustainable way, not only from an economical point of view. Emergencies need a biomedical approach; complex illnesses and the huge burden of chronic diseases need a complex approach that needs to be broadened with the person-centred vision of TCAM systems for a social and epistemological reformulation of *medicine*.

### Broadening health and healing

In a person-centred, salutogenetic context, the concept of health needs to be broadened. Health is more than the absence of illness. It has been described as a complete state of physical, mental and social wellbeing [14]. However, this definition brings a static and even utopic element to the concept of health that can appear abstract and far away from the daily realities of human life. Challenges are a part of life and illness is a challenge to our bio-psycho-spiritual integrity. We live in the constant pursuit of a subtle and mobile equilibrium between health, illness and healing. According to salutogenetic principles, it is not the absence of hardship or illness that determines health, but our ability to deal with them positively, with the confidence to face them, with the knowledge that we can rely on ourselves to overcome them and with the trust that these difficult events hold meaning for our lives. A dynamic concept that reflects what we live through daily in different forms is introduced to the concept of health. Health becomes the ability to adjust and self-manage. The pursuit of health and the process of healing become an active process of continuous adjustment between our physiological, psychological and spiritual integrity, and the outer or inner influences that can strengthen or undermine this [15].

Healing is a broader concept than curing. According to Jonas, ‘healing is the process of recovery, repair and the return to wholeness, in contrast with “curing” which focuses on the eradication of disease. While mainstream health care has traditionally operated from a “cure” model, the time has come to create a new model of health care delivery that makes room for both healing and cure’ [16]. Returning to ‘wholeness’ implies a process that concerns not only physical aspects, but also psychological and spiritual ones. It is a process that takes time. It requires active involvement on the part of the whole person with the help and guidance of the caregiver. The element of change over time has an evolutionary quality: going through experiences that challenge the essence of our being and finding ourselves again, or ‘returning to wholeness’; we are not the same as we were before we started.

This attempt to unite healing and cure is central to the future development of medicine. At the core of this are people: patients, doctors and health-care professionals in general. It is interesting to note how, in policy and research terminology, we are far away from this. ‘Physicians’ become ‘providers’, and ‘patients’ become ‘consumers’ or ‘clients’ [17]. This is not just a question of nominalism but is symptomatic of a deeper change. We understand the need for objective research, for parameters that will allow the rational distribution of limited resources and for guidelines to inform clinical decisions and ensure a basic and uniform standard of care. In this attempt, however, evidence-based medicine (EBM) has become too rigid and impersonal. When randomised controlled trials (RCTs), the most impersonal research method available, are considered the only form of evidence that can influence regulatory decision-making, this shows a progressive change away from the core of the medical act which is a meeting between people: the physician (and other caregivers, each with their competencies) and the patient. ‘Healing’ and ‘disease alleviation’ are the tasks of medicine; they are not ‘primary outcome measures’. The risk of having an impersonal research method to guide and evaluate care is that the abilities of the art of treatment and of empathic engagement continue to wither away [18,19]. ‘Doctors lose the ability to heal’. By applying guidelines, they become reliant on controlling disease processes with drug progressions or repeating the same tests sequentially to detect when another medication may be needed [10]. The medical act needs to be placed at the centre of medicine. The key to a successful and satisfying therapeutic process centres on the meeting between human beings: a physician, or other caregivers, and a patient, who work together towards healing. Establishing participatory medicine [20] is important to end paternalism in the doctor-patient relationships [21]. Patients are increasingly informed and need to be informed about their care. The consultation needs to be based on the encounter between individuals as equals, each with their competencies. Physicians and health-care professionals bring their professional, technical and caring skills; patients bring knowledge of their illness, the experience of their discomfort and their life.

‘Patient-centered medicine is, above all, a metaphor. “Patient centred” contrasts with “doctor centred” and replaces a Ptolemaic universe revolving around the physician with a Copernican galaxy revolving around the patient. The flaw in the metaphor is that the patient and the doctor must coexist in a therapeutic, social, and economic relation of mutual and highly interwoven prerogatives. Neither is the king, and neither is the sun. Health relies on collaboration between the patient and the doctor, with many others serving as interested third parties. Patient and physician must therefore meet as equals, bringing different knowledge, needs, concerns, and gravitational pull but neither claiming a position of centrality. A better metaphor might be a pair of binary stars orbiting a common centre of gravity, or perhaps the double helix, whose two strands encircle each other, or—to return to medicine's roots—the caduceus, whose two serpents intertwine forever’ [22].

### Decision-making and evaluation in person-centred health care

There are many studies and papers that underline the importance of patient experiences, patient preferences and patient-based outcomes to guide therapeutic choice. The concept of goal-oriented care complements a disease outcome-based paradigm of care where managing diseases as well as possible, according to guidelines and population goals, is valued more than asking what patients want. Doing what is right for the patient takes on a central role in the future of medicine. Any evaluation of success in complex, chronic situations must, above all, consider patients' preferred outcomes.

In goal-oriented care, the most important signs and symptoms to evaluate conditions like heart failure or COPD are those connected with carrying out daily activities that are important and meaningful to the patient. This can be dyspnoea in walking their grandchild to school, rather than in the context of a generalised list of symptoms benchmarked against disease-specific outcomes. An elderly patient with hypertension and postural hypotension may decide not to take blood pressure-lowering medication in order to be able to walk with less fear of falling [23]. Together with our patients, after having explained the meaning and the implications of a choice, we may decide that an improvement in being able to carry out daily life activities is more important than lowering blood pressure to prevent a potential stroke. For another patient, the reverse may be true.

**Figure 1:**
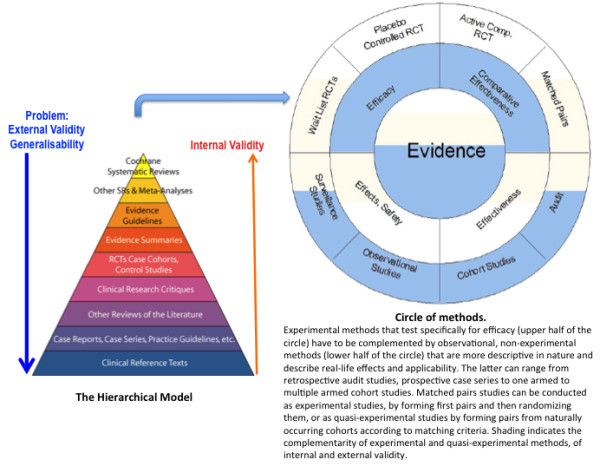
**From a hierarchical to a circular model of evidence (Adapted from**[26]**).**

PCM needs to be founded on sound clinical judgment. A cluster RCT looked at patients with type 2 diabetes and hypertension. One group was treated according to guidelines, the other at the physician's discretion. They noticed that there was no difference in blood pressure control after 1 year. However, the guideline group was more likely to receive higher doses of antihypertensive drugs and had consulted the physicians significantly more often [24]. This brings evidence towards the importance of clinical judgment in individualising treatments that are suited to a particular person. This can reduce the amount of drugs prescribed and the dose required with a consequent reduction in costs and a better quality of life for the patient. Clinical judgment needs to be developed alongside a good understanding of EBM during training and needs to form an important part of continued professional development. The method of cognition-based medicine [25] can give additional research tools for the scientific evaluation of individualised interventions. To investigate complex interventions in all their aspects and their context, a circular model of evidence can be used (Figure 1) [26]. The hierarchical model of EBM holds good internal validity, but this does not necessarily correlate with external validity. It is also difficult to translate it to practical clinical life when evaluating complex clinical situations or complex medical systems like TCAM systems. A circular model of evidence relies on the complementarity of the research methods that balance respective strengths and weaknesses. There is no privileged vantage point from which to define truth. This is a strategy to evaluate complex interventions that can add investigative tools to PCM and to the pluralism of thorough but open-minded scientific research [26].

### The educational task of person-centred medicine

Person-centred medicine has the educational task to facilitate professional development in the directions described above, for each professional role, from as early as undergraduate training. Training should include courses and exercises in epistemology, holistic or multi-perspective thinking to enable the development of independent thought. The development of agile thinking abilities that are able to conceptualise changing processes can be achieved through the exercise of artistic activities practised according to specific methods.

Health-care professionals need to be equipped with knowledge and competencies that will enable them to work efficiently in health-care systems where organisational assets are changing across the world. This is particularly important if they hold managerial positions. All over the world, health-care systems are falling in line with each other. Practice and procedures are becoming standardised with a tendency towards centralisation of services into regional centres of excellence. Increasing importance is also being placed on TCAM systems. Current and future health-care professionals need to acquire knowledge about national health-care systems and about national health-care structure networks as well as an understanding of how the rest of the world's health-care systems work. This will enable them to deal with new pandemic diseases and chronic and invalidating illnesses and to develop measures for health promotion at a global level. New competencies are needed to provide care to migrant patient populations, many of whom come from cultures with specific and different medical approaches. Knowledge and skills are also necessary to meet the growing demand from citizens who wish to be able to choose treatment pathways, interventions and health-care practices that come from TCAM systems.

During their undergraduate and postgraduate education, health-care professionals need to be given the tools to understand the social changes that are taking place, including in the health-care system, and to address them with adequate sanitary responses and coherent political proposals [27]. People with decision-making power at all levels of the welfare system, of health-care organisations and of organisations in the public sector, free market or tertiary sector that provide services aimed at improving wellbeing are being called upon to give new solutions regarding health and health care. Current and future managers need to develop the skills that will enable them to plan, achieve and manage socio-sanitary initiatives in an innovative way, developing the public sphere with a system that considers the *person* as the core element and which aims towards health and wellbeing.

In order to have a person-centred approach, technological proficiency, solid grounding in EBM and knowledge of international protocols need to be complemented by knowledge of the epistemological systems and practical tools of TCAM. Doctors and other health-care workers need to be taught clinical judgment to become an accurate decision-making tool [28]. This complements the application of EBM and protocols, which are based on large numbers and therefore not always applicable to particular cases. Clinical judgment allows personalisation of guidelines and protocols to each case. It leads to more accurately placed resources and less waste. Learning and teaching about salutogenetic practices means that health-care workers may adopt healthier lifestyles for themselves. Greater work satisfaction and active involvement strengthen a health-care worker's resilience and sense of coherence, ultimately improving his or her health as well as that of his or her patients [15].

### Developing salutogenetic health care

TCAM systems emphasise all aspects that influence the choice of lifestyle, the overall situation, patient outcome, therapy response, wellbeing and treatment compliance. These depend on multiple and interacting cofactors such as correct nutrition, regular exercise, adequate rest, biographical features, family, socioeconomics, health inequalities, the social gradient of health, the gender gradient, coming from a particular cultural or religious background and having a particular spiritual vision of life [17]. Although some TCAM caregivers have lost the link with the spiritual dimension of their discipline, traditionally this forms an essential part of the philosophical background that underpins TCAM systems. With variations, the human being is seen as the bearer of a sacred, incarnated spiritual element. The pursuit of health in all its aspects is instrumental to allow the human being to unfold his or her incarnation and aims in this life.

Lifestyle changes form an essential, cost-effective part of generating health, preventing disease, treating illness and reducing mortality. They are also important in giving people responsibility for maintaining their own health. In a person-centred approach, lifestyle intervention needs to take into consideration all aspects of the human being and be personalised by tailoring it to the individual. Health promotion is a form of disease prevention. The first topic to be addressed in health promotion and health education is diet and the quality of foodstuffs. If diet were a better used resource in health care and people were taught its principles and practical applications, this may decrease the need to use medication in some cases. The care of biorhythms is another aspect to be addressed. Altered sleeping patterns, eating rhythms and patterns of physical activity are both a measure and a cause of pathology and health. The benefits of regular exercise are well known to everyone. It is important for people to become aware of their own biorhythms, which alter with age, gender, state of health and personal and constitutional characteristics. With the help of education, improved environmental conditions and appropriate care during illness, people can adjust or at least compensate for altered biorhythms and therefore actively improve their health. The emotional factors that contribute to health and illness can also be addressed. Negative emotions increase the incidence of physical illness. Conversely, positive emotions and humour improve health and wellbeing. Chronic stress can strengthen or weaken a person depending on how strong or weak his or her resilience and sense of coherence are [15,29]. Chronic stress and social deprivation have been shown to be similar risk factors for developing ischaemic heart disease compared to hypertension or hypercholesterolaemia [30]. Emotions can have adverse effects on the incidence of complication and prognosis during an acute cardiac event. Emotional resilience and SOC can be strengthened through artistic activity in the form of painting, music, dance, drama and creative writing. Through artistic activities, people learn new skills and their achievements have a positive effect on their self-esteem. This can be applied to overcome future challenges or unknown life situations with strengthened sense of coherence [15].

It has been shown that in some areas, we can obtain better therapeutic results with lifestyle changes compared to drug therapy. The influence of these factors on widespread disease such as diabetes, hypertension and coronary disease is immense and, in some areas, larger than the effects of drug treatments [30-33]. The INTERHEART study showed a more than 90% reduction in the risk of developing MI by adopting a healthier lifestyle, whereas high-cost interventional procedures do not show additional benefits in RCTs [34]. Another study in a cohort of apparently healthy men showed how adherence to healthy lifestyles was associated with lower risk of developing heart failure later in life [35].

To induce sustainable motivation for change, we cannot simply inform patients about risk factor reduction, which can be experienced as boring or abstract, or risk of death, which can frighten patients and therefore block off their further listening. The challenge is to develop individualised therapy concepts to activate the person's own resources considering his or her potential, values and environment. Strategies of lifestyle change must be tailored to the individual patient in order to be feasible and to cause him or her to quickly feel better and satisfied [17]. This is the key to long-term compliance. The other core aspect is human contact. Any lifestyle change requires questioning of values and sometimes deeply engrained habits. It is difficult to change them; there will be frustration, rebellion, refusal and relapse. The physician or caregiver needs to have the tools to deal with this, taking time to support patients at these times, encouraging and advising, and providing additional therapies if necessary. Giving information leaflets can be informative, but it cannot replace the importance of the doctor-patient relationship, which requires competence, patience and a profound conviction on the part of the physician or health-care professional.

### Developing a therapeutic relationship based on empathy

The clinical encounter, born out of a more or less explicit request for help on part of the patient, is a chance to develop a therapeutic relationship. This is not an automatic transition. The time for paternalism may be over, but merely giving information, personally, through leaflets or computer-based systems is only part of the answer. It is through empathy that the therapeutic relationship can be established. Generically, empathy is defined as the ability to understand and share the feelings of another [36]. Edith Stein goes even further to describe empathy as the way in which we perceive the experiences lived by another human being. Feelings become qualified as a tool for perception, like judgment which is a tool for cognitive understanding. ‘Other human beings are not given to my perception as physical bodies, but as a sensitive, living body belonging to an “I”. An “I” that senses, thinks, feels and wills. The living body of this “I” not only fits into my world of phenomena but it is itself the orientation point of such a world of phenomena. It faces this world and communicates with me. The other human being is another living being, structurally akin yet foreign to me. Yet I can perceive his or her experiences through empathy’ [37]. This adds an element of understanding a deeper cognitive process to the state of sympathy, where we are purely feeling what another person feels without discernment [38].

Empathy does not mean feeling the happiness or sorrow of another human being. It means widening our experience, enabling it to comprehend another person's joy or sorrow and maintaining the distinction between our self and the other. ‘Empathy calls on the essential aspect of creating a relationship: going from the continuity of being one with another person, to the contiguity of being with the person’ [39].

Empathy does not cloud clinical judgment; on the contrary, it improves the latter because the underlying feeling is one of understanding in the broader sense. Empathy does not necessarily make the clinical encounter longer. There is research to suggest the opposite [40] because developing empathy allows the caregiver to grasp the core of the problem more quickly; this includes how the patient experiences illness as well as the subtle and contextual causes that have contributed to it. This in turn is the basis on which individualising advice and therapies can occur.

Empathy is a quality that is present in those with a ‘flexible, mature and well-established personality’ [41]. Stein goes further to say that ‘empathy is possible if there is a fundamental correspondence between my being and the being of the other, if the “typus” is the same. However, as spiritually every person is a unique “typus”, I will only be able to empathise with another person by the degree in which I have also become a “person”, a totality, a whole with a purpose and meaning. Only then can I hope to understand another person. Otherwise we close ourselves off in our peculiar nature and other people become foreign to us or worse, we model them on our image distorting reality’ [37].

Empathy requires training during higher education and continuous development during professional life in order to grow and become more skilfully used, but empathy cannot be identified with a particular habitus and even less as a communication technique. It is a way of being, of ‘being available’, which has its structure in the ontological and cognitive structures of the human being.

Educating ourselves to empathy as physicians means keeping our perception always alert, not letting ourselves being carried away by the illusion that knowledge of the patient's physical condition automatically means knowledge of his or her personal experience and of his or her personal reality. It means taking healing and cure back to their original source where they are services and not merely interventions or outcomes, in which they are answers before they are questions. It is the ability *to be*, which comes before and above the ability *to do*[39].

TCAM systems give practical, cognitive and meditative tools to physicians and caregivers to better develop these aspects. MCP requires that empathy be continuously renewed, deepened and refined for the good of patients and health-care professionals alike. Empathy adds a spiritual element to medicine because it intrinsically includes the most authentically human experience there is: that is the encounter between people. Through empathy, we perceive the other person in his or her intrinsic value, with his or her world of values, not because he or she does good in the world, but because his or her existence is intrinsically valuable to the world.

Studies that evaluate the success of lifestyle changes as therapeutic interventions to improve illness show that the main reason for failure is maintenance over time. For example, diet schemes, with a lot of human contact, are as good as bariatric surgery for treating obesity in some cases, but bariatric surgery shows better results in maintaining weight loss at long-term follow-up several years later. Lifestyle changes are difficult to undertake; they require active involvement to challenge habits and beliefs. A therapeutic relationship based on empathy, which has the flexibility to be continued over long periods of time, gives more opportunities for these interventions to be successful. A therapeutic relationship based on empathy can be cost-effective [40], improve adherence to treatment plans [42] and enhance patient health outcomes [43].

## Conclusions

### Recommendations for the future development of person-centred medicine

PCM needs to be developed by broadening biomedicine with the epistemological basis, the diagnostic and therapeutic tools of TCAM systems, which stems from citizens, patients and health-care professionals alike. In this process, a balance needs to be struck between overcoming global health inequalities and individualising care.

TCAM systems offer a vision that considers the human being in his or her bio-psycho-spiritual aspects. They broaden the paradigm of personalised, preventive and predictive medicine. They are inclusive and participatory medical systems.

Personalisation in medicine needs to consider the individual as a unique being where the whole is more than the sum of its parts. Prediction needs to take into consideration dynamic understanding of the relationship between the physical, psychological and spiritual aspects of the human being, which allows the identification and treatment of pathological tendencies before they become overt organic disease.

Prevention needs to include health education and salutogenetic interventions aimed at all aspects of the human being that give patients the tools to take better responsibility for their own health. This has long-term effects and a low economic impact for the development of more sustainable health care. The medication and non-medication therapies of TCAM systems are also salutogenetic by strengthening physiological innate self-healing abilities. They complement the symptomatic and disease-based approach of biomedical interventions.

The basis to develop salutogenetic lifestyle changes that can last over time or change according to changing circumstances needs to be a therapeutic relationship based on empathy. TCAM systems can give the practical, epistemological and meditative tools to develop this. A therapeutic relationship based on empathy is a requirement for developing true participatory medicine.

PCM needs evaluation tools that go beyond EBM to include a circular model of evidence to evaluate the complexities of TCAM systems. The method of cognition-based medicine can give additional research tools for the scientific evaluation of individualised interventions. Patient-based outcomes need to become a core aspect of clinical evaluation. Health-care education needs to be broadened accordingly to give doctors and health-care workers of the future the tools to act in innovative and highly differentiated ways, always guided by deep respect for individual autonomy, personal culture, religion and beliefs.

## Competing interests

The authors declare that they have no competing interests.

## Authors’ contributions

The authors worked together on the article, reflecting on the paradigm, planning the article and writing it. All authors read and approved the final manuscript.

## Authors’ information

PRdS is the Expert for Non-conventional Medicine in the High Council of Health, Ministry of Health, Italy; the President of the Charity Association for Person Centred Medicine, Bologna, Italy; and the Director responsible for Complementary and Alternative Medicine and Contacts to Patient Organisations, National EPMA Board in Italy. MA is the Vice President of the Charity Association for Person Centred Medicine, Bologna, Italy, and the former President of the Italian Society of Anthroposophic Medicine (SIMA). PG is affiliated to the Charity Association for Person Centred Medicine, Bologna, Italy, and to the SIMA.
